# Rising challenge of multidrug-resistant tuberculosis in China: a predictive study using Markov modeling

**DOI:** 10.1186/s40249-020-00682-7

**Published:** 2020-06-08

**Authors:** Bing-Ying Li, Wen-Pei Shi, Chang-Ming Zhou, Qi Zhao, Vinod K Diwan, Xu-Bin Zheng, Yang Li, Sven Hoffner, Biao Xu

**Affiliations:** 1 School of Public Health, Fudan University, Shanghai, China; 2 Key Lab of Health Technology Assessment (Fudan University), National Health Commission, Shanghai, China; 3 Department of Cancer prevention, Fudan University Shanghai Cancer Center, Shanghai, China; 4 Department of Public Health Sciences (Global Health/IHCAR), Karolinska Institutet, Stockholm, Sweden; 5 Department of Nephrology, Zhongshan Hospital, Fudan University, Shanghai, China

**Keywords:** Multidrug-resistant tuberculosis, Markov chains, Prevalence, Prevention and control

## Abstract

**Background:**

Multidrug-resistant tuberculosis (MDR-TB) is on the rise in China. This study used a dynamic Markov model to predict the longitudinal trends of MDR-TB in China by 2050 and to assess the effects of alternative control measures.

**Methods:**

Eight states of tuberculosis transmission were set up in the Markov model using a hypothetical cohort of 100 000 people. The prevalence of MDR-TB and bacteriologically confirmed drug-susceptible tuberculosis (DS-TB^+^) were simulated and MDR-TB was stratified into whether the disease was treated with the recommended regimen or not.

**Results:**

Without any intervention changes to current conditions, the prevalence of DS-TB^+^ was projected to decline 67.7% by 2050, decreasing to 20 per 100 000 people, whereas that of MDR-TB was expected to triple to 58/100 000. Furthermore, 86.2% of the MDR-TB cases would be left untreated by the year of 2050. In the case where MDR-TB detection rate reaches 50% or 70% at 5% per year, the decline in prevalence of MDR-TB would be 25.9 and 36.2% respectively. In the case where treatment coverage was improved to 70% or 100% at 5% per year, MDR-TB prevalence in 2050 would decrease by 13.8 and 24.1%, respectively. If both detection rate and treatment coverage reach 70%, the prevalence of MDR-TB by 2050 would be reduced to 28/100 000 by a 51.7% reduction.

**Conclusions:**

MDR-TB, especially untreated MDR-TB, would rise rapidly under China’s current MDR-TB control strategies. Interventions designed to promote effective detection and treatment of MDR-TB are imperative in the fights against MDR-TB epidemics.

## Background

Tuberculosis (TB), once called the “white plague”, remains one of the leading causes of deaths from infectious diseases. In 2017 alone, TB caused 10.0 million incidents and resulted in 1.3 million deaths worldwide [1]. Over the past two decades, however, both the incidence and mortality of TB have decreased remarkably thanks to the global efforts against TB. Regardless, drug-resistant tuberculosis, especially multidrug-resistant tuberculosis (MDR-TB), remains a major obstacle in the task of putting an end to the global TB epidemic [2, 3]. The most recent World Health Organization (WHO) report suggests that MDR-TB and rifampicin-resistant tuberculosis (RR-TB) together accounted for 558 000 incidents and 230 000 deaths in 2017 [1]. In the meantime, numerous countries across the world have witnessed a growth in the prevalence of MDR-TB over time, which poses a greater threat to the global prospects of TB control [4, 5].

Given the high priority to TB control, several mathematic models have been developed to estimate the burden of TB [6–9]. Although a few models have projected the burden of TB in different countries, few gave MDR-TB a specific estimation [10, 11]. Among the proposed forecast models, Markov process is a widely used mathematical stochastic process with the capability to simulate the progress of disease according to certain probabilistic manner between different states [12–14].

China has the second highest case burden of MDR-TB worldwide. In 2017, China had an estimated 58 000 MDR-TB/RR-TB incidents, accounting for approximately 10% of the global burden. However, only 22.5% of the above-mentioned estimated MDR-TB/RR-TB cases were laboratory-confirmed and only 10.2% started on treatment, presenting a much lower MDR-TB detection rate and treatment coverage compared to the global average [1]. In the past decades, although China has made remarkable successes in TB control, these achievements might be compromised by the rising prevalence of drug resistance. Illustrating the trajectory of MDR-TB burden will provide a full view of China’s TB epidemics and give insights on policy and strategy development in line with the End TB Strategy, which aims to reduce global TB deaths by 95% and incidence by 90% by the year of 2035 [15]. As the main source of infection transmission, bacteriologically confirmed TB plays a key role in tuberculosis control. Herein, a dynamic Markov model is constructed based on the prevalence of bacteriologically confirmed TB in the national survey to predict the prevalence of MDR-TB and bacteriologically confirmed drug-susceptible TB (DS-TB^+^) longitudinally under the current China TB/MDR-TB epidemics; and to estimate possible gains from the enhancement of MDR-TB case finding and treatment coverage by 2050.

## Methods

### Model approach

**Fig. 1:**
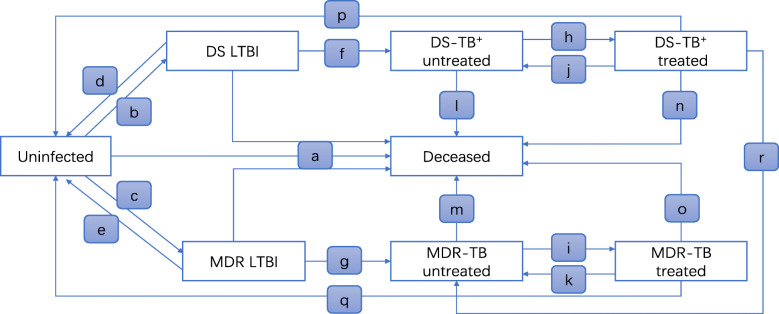
Structure of the mathematical model. LTBI, latent tuberculosis infection; DS-TB^+^, bacteriologically confirmed drug-susceptible TB; MDR-TB, multidrug resistant tuberculosis

We constructed a population-level, dynamic and compartmental Markov model in which individuals are grouped into mutually exclusive compartments based on the epidemiological characteristics of TB infections and early model studies [7, 8]. The model, subdivided into MDR-TB and DS-TB^+^, explicated the occurrence of TB transmission, the development from latent tuberculosis infection (LTBI) to active TB, anti-TB treatment, and treatment outcomes. It simulated the transition among eight states: (1) uninfected; (2) latent infection of drug-susceptible tuberculosis (DS LTBI); (3) latent infection of MDR-TB (MDR LTBI); (4) DS-TB^+^ untreated; (5) MDR-TB untreated; (6) DS-TB^+^ treated; (7) MDR-TB treated; (8) deceased (defined in Fig. 1). These eight states were described in Table 1 while the eighteen parameters (from a to r) for transmission of states were presented in Table 2. If patients didn’t receive the recommended treatment, TB was considered untreated. HIV was not explicitly considered because of the low percentage of TB/HIV co-infection (roughly 1%) in China [1].

### Model assumptions

1. Individuals recovered from active TB disease are assumed to have the same risk of infection as the uninfected population [26, 27].
2. The possibility of moving from DS-TB^+^ untreated to MDR-TB untreated was not considered because the treatment coverage of DS-TB reached nearly 90% in China [1].
3. Individuals with active TB disease cannot be re-infected before recovering from the current disease episode [26].

### Model initialization and parameter setting

Simulations of the Markov model were initiated in 2011 for the use of the Fifth National Tuberculosis Epidemiological Survey in 2010, the most recent survey with a sample size of 252 940 by random sampling [16]. At the base year of modeling, a hypothetical cohort of 100 000 Chinese was established and divided into eight TB states. Based on the major surveys in China, the prevalence of LTBI and DS-TB^+^ were 20% and 66/100 000 respectively, and the proportion of MDR-TB accounted for 6.8% of the total TB cases [16–18]. The description of each state, the initial data set and data sources were listed in Table 1.

**Table 1:** State descriptions and initial data set among the cohort of 100 000 people

	Description	Population at start	Sources
Uninfected	Individuals without TB infection (latent or active)	79 948	Wang LX [16]
DS LTBI	Individuals with latent infection of DS-TB	18 627	Wang LX [16], Gao L [17], Li X [18]
MDR LTBI	Individuals with latent infection of MDR-TB	1359	Wang LX [16], Gao L [17], Li X [18]
DS-TB^+^ untreated	Active DS-TB^+^ cases not treated with TB regimens	62	Wang LX [16]
MDR-TB untreated	Active MDR-TB cases not treated with MDR-TB regimens	4	Wang LX [16]
DS-TB^+^ treated	Active DS-TB^+^ cases undergoing treatments	0	Starting from 2012 (the second cycle of the model)
MDR-TB treated	Active MDR-TB cases undergoing MDR-TB treatment	0	Starting from 2012 (the second cycle of the model)
Deceased	Deaths from 100 000 population	0	Starting from 2012 (the second cycle of the model)

*DS* drug-susceptible; *TB* tuberculosis; *DS-TB* drug-susceptible tuberculosis; *LTBI* latent tuberculosis infection; *MDR* multidrug-resistant; *MDR-TB* multidrug-resistant tuberculosis; *DS-TB*^*+*^ bacteriologically confirmed drug-susceptible tuberculosis

Transition between states depends on the parameters which determine the pathway of movement within the model in each yearly cycle. Eighteen parameters were defined and extracted from national annual statistics or TB registration, and the estimation of parameters with uncertainty was based on the WHO TB database, the WHO annual report on TB, and published literature [1, 17, 19–25]. Most of these parameters were held fixed throughout our analysis, except for b and c, which varied with TB prevalence. Table 2 shows details on these probabilities.

**Table 2:** Markov model probabilities input and sources

	Description/Explanation	Value	Source
a	Probability of dying among Uninfected or LTBI	0.007	National Bureau of Statistics of China [19]
b	Probability of acquiring DS LTBI from being Uninfected, varies with DS-TB^+^ prevalence. b = (t_1_n_1_ + t_2_n_2_)/N_0_, where t_1_ is the number of ‘DS-TB^+^ treated’, n_1_ is the number of infections each ‘DS-TB^+^ treated’ person causes, t_2_ is representing ‘DS-TB^+^ untreated’ cases, n_2_ is the number of infections each ‘DS-TB^+^ untreated’ person causes, and N_0_ is the total number of the ‘Uninfected’.	Initial value is 0.003	Assumed based on finding from WHO [1], Huang LQ [20], and Sun CF [21]
c	Probability of acquiring MDR LTBI from being Uninfected, varies with MDR-TB prevalence. c = (t_3_n_3_ + t_4_n_4_)/N_0_, where t_3_ is the number of ‘MDR-TB treated’, n_3_ is the number of infections each ‘MDR-TB treated’ person causes, t_4_ is representing ‘MDR-TB untreated’ cases, n_4_ is the number of infections each ‘MDR-TB untreated’ person causes, and N_0_ is the total number of the ‘Uninfected’.	Initial value is 0.0005	Assumed based on finding from WHO [1], Huang LQ [20], and Sun CF [21]
d	Probability of clearing MTB infection for DS LTBI persons	0.05	Xu KJ [22]
e	Probability of clearing MTB infection for MDR LTBI persons	0.05	Xu KJ [22]
f	Probability of acquiring active DS-TB^+^ from LTBI	0.004	Assumed based on finding from Gao L [17], Mandal P [23], and Xu J [24]
g	Probability of acquiring active MDR-TB from LTBI	0.004	Assumed based on finding from Gao L [17], Mandal P [23], and Xu J [24]
h	Probability of acquiring treatment for DS-TB^+^	0.87	WHO [1]
i	Probability of acquiring treatment for MDR-TB	0.10	WHO [1]
j	Probability of treatment failure and loss to follow up for DS-TB^+^	0.04	WHO tuberculosis database [25]
k	Probability of treatment failure and loss to follow up for MDR-TB	0.13	WHO tuberculosis database [25]
l	Probability of dying among untreated DS-TB^+^	0.10	Assumed based on finding from WHO [1]
m	Probability of dying among untreated MDR-TB	0.40	Assumed based on finding from WHO [1]
n	Probability of dying among treated DS-TB^+^	0.01	WHO tuberculosis database [25]
o	Probability of dying among treated MDR-TB	0.04	WHO tuberculosis database [25]
p	Probability of treatment success for DS-TB^+^	0.93	WHO tuberculosis database [25]
q	Probability of treatment success for MDR-TB	0.41	WHO tuberculosis database [25]
r	Probability of acquired drug resistance from DS-TB^+^ to MDR-TB	0.014	WHO tuberculosis database [25]

*LTBI* latent tuberculosis infection; *DS* drug-susceptible; *MDR* multidrug-resistant; *TB* tuberculosis; *MTB Mycobacterium tuberculosis*; *DS-TB*^*+*^ bacteriologically confirmed drug-susceptible tuberculosis; *MDR-TB* multidrug-resistant tuberculosis

### Modeled scenarios

Using the model, we projected the impact of three hypothetical scenarios on the epidemics of MDR-TB, including 1) improving detection rate; 2) expanding treatment coverage; 3) increasing detection rate and treatment coverage. Detection rate was the number of diagnosed MDR-TB cases divided by the estimated TB cases. Treatment coverage was expressed as the percentage of notified MDR-TB patients who commenced the WHO recommended MDR-TB treatment. In scenario 1, detection rate was assumed to increase to 50% or 70% at 5% per year from 2020 (baseline is 22.5%). In scenario 2, treatment coverage of MDR-TB was anticipated to reach 70% or 100% at 5% per year from 2020 (baseline is 45.5%). In scenario 3, both detection rate and treatment coverage were assumed to be 70% with a 5% annual increase from the baseline.

### Model outcomes

We predicted TB prevalence trends from 2019 to 2050, under the current TB control conditions and three intervention schemes. The prevalence of treated MDR-TB was defined as the number of cases enrolled in MDR-TB treatment per 100 000 people. Untreated MDR-TB prevalence was the number of MDR-TB cases left untreated per 100 000 people. The effect of the above-mentioned three scenarios was measured via reduced MDR-TB prevalence compared with the baseline.

### Statistical analysis

Tree Age Pro 2011 software (Tree Age Software, Inc., Williamstown, MA, USA) and Microsoft Excel 2016 (Microsoft Corporation, Redmond, WA, USA) were used to conduct the Markov chain models. GraphPad Prism 6.0 (GraphPad Software, Inc., La Jolla, CA, USA) was used to draw the figures.

## Results

### TB epidemic trends providing no intervention changes to current TB control conditions

**Fig. 2:**
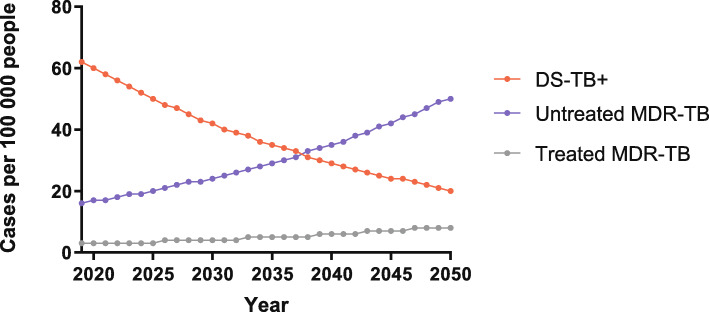
Predicted TB prevalence from 2019 to 2050 under current TB control conditions. DS-TB^+^, bacteriologically confirmed drug-susceptible TB; MDR-TB, multidrug-resistant tuberculosis

Without any intervention changes to the current conditions, the model projected a considerable decrease in DS-TB^+^ prevalence and a substantial increase in MDR-TB prevalence from 2019 to 2050. In this case, treated and untreated DS-TB^+^ were not separated considering the high treatment coverage of DS-TB^+^ in China. It was estimated that, by 2050, DS-TB^+^ prevalence would decline to 20 per 100 000, demonstrating a 67.7% reduction from 2019. Conversely, MDR-TB prevalence would increase by three times approximately from 19/100 000 in 2019 to 58/100 000 in 2050. It was further estimated that the prevalence of untreated MDR-TB would increase from 16/10 000 in 2019 to 50/100 000 in 2050, whereas a limited increase would be seen in treated MDR-TB, resulting in a prevalence of 8/100 000 in 2050. By the year of 2050, 74.4% of TB cases would be MDR-TB, 86.2% of which would remain untreated. These untreated MDR-TB cases would account for most TB cases and remain the main reason behind the aggravation of the ongoing TB epidemic. Figure 2 discloses in details the simulation results of the model under baseline condition.

### MDR-TB epidemic trends with increased detection rate or treatment coverage

**Fig. 3:**
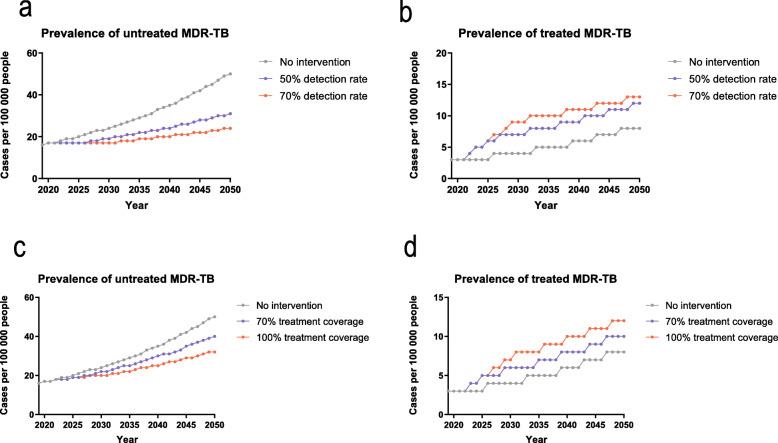
MDR-TB prevalence from 2019 to 2050 under different detection rate or treatment coverage. **a** untreated and **b** treated MDR-TB prevalence with increased detection rate; **c** untreated and d) treated MDR-TB prevalence with increased treatment coverage. MDR-TB, multidrug-resistant tuberculosis

Scenario 1 presented detection rate of MDR-TB increased to 50% or 70% at 5% per year, leading to a projected MDR-TB prevalence with an upward trend much slower than that of the baseline. Under the same scenario, MDR-TB prevalence was estimated to be 43/100 000 or 37/100 000 by 2050, with a detection rate of 50% or 70%, respectively (Fig. 3). Compared to the baseline, MDR-TB prevalence displayed a predicted reduction of 25.9 and 36.2% respectively, whereas 64.9% of MDR-TB cases would remain untreated with the recommended MDR-TB regimens even with a 70% detection rate.

With MDR-TB treatment coverage improved to 70% or 100% at 5% per year as indicated in Scenario 2, MDR-TB prevalence was estimated to be 50 or 44 per 100 000 people by 2050, with a treatment coverage of 70% or 100% (reduced by 13.8 and 24.1%), respectively (Fig. 3). whereas 72.7% of MDR-TB cases would remain untreated due to being undetected even if with a treatment coverage of 100%.

### TB epidemic trends with simultaneously increased detection and treatment coverage

**Fig. 4:**
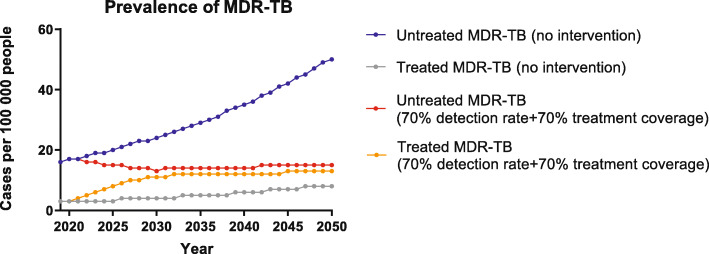
MDR-TB prevalence from 2019 to 2050 under Scenario 3. MDR-TB, multidrug-resistant tuberculosis

In scenario 3 where both detection rate and treatment coverage were set to be 70% with an annual increase of 5% from baseline, the prevalence of MDR-TB would be reduced remarkably and was estimated to reach 28/100 000 or lower, demonstrating a 51.7% decrease compared to the baseline. And the proportion of untreated cases would be 53.6% by 2050. Figure 4 shows the trends of MDR-TB in Scenario 3 compared with the baseline.

## Discussion

A Markov model was constructed in this study to forecast trends of DS-TB^+^ and MDR-TB in China—a country with heavy burden of TB/MDR-TB. It was estimated that, if the present TB control measures remained unchanged, DS-TB^+^ prevalence would see a significant decrease, but the MDR-TB epidemic would grow at an alarming rate, indicating a gradual shift in the TB epidemics in China from drug-susceptible tuberculosis (DS-TB) to MDR-TB. In addition, most MDR-TB cases would remain untreated, and thus would continue to transmit the disease. The results suggested that increasing detection rate of MDR-TB could have an obvious effect on MDR-TB prevalence but could not interrupt the emerging trend of MDR-TB prevalence. Meanwhile, with the present MDR-TB detection rate in China being significantly low, MDR-TB prevalence would continue to increase even if treatment coverage improved to 100%. Therefore, detection rate must be enhanced alongside treatment coverage to achieve a significantly reduction in MDR-TB prevalence.

A Markov model was built in the study that separated TB prevalence into DS-TB and MDR-TB sub-categories to achieve in-depth observations and detailed predictions for the TB epidemic in China. The projected DS-TB^+^ and MDR-TB prevalence was consistent with those projected in previous studies [28, 29], indicating that MDR-TB prevalence would continue to rise. In addition, simulations suggested that most MDR-TB cases would remain untreated, illustrating the urgency for improving MDR-TB detection and treatment. In the meantime, this study constituted as first step to better understanding the trends of both untreated and treated MDR-TB in China and the outcome is also applicable for other high-burden, low-to-middle income countries.

Increasing MDR-TB detection rate from 22 to 70% is challenging but achievable. The low MDR-TB detection rate resulted from a combination of issues, including a lack of laboratory capacity for drug susceptibility test (DST), insufficient funding and inequity in health financing, high cost associated with rapid diagnostics, and low clinical alert for TB [30]. Under China’s current TB control program, health financing, health insurance and health care for MDR-TB are localized and determine the elementary medical services and expenses. The lack of DST resulted in low case detection in some TB-designated health facilities on the county and district level, who in return had to deliver sputum samples to upper-level facilities for DST [31]. In addition, restricted by resources and the capacity in MDR-TB health care, drug-resistance testing is only recommended to patients at high risk of MDR-TB under China’s current MDR-TB control program, whereas these patients merely account for less than 50% of all MDR-TB cases [30]. Even when DST is available, the results can take months to come back, meanwhile, patients would have already been treated with DS-TB regimen [32]. Unfortunately, the use of rapid molecular diagnostics is limited due to their costs and the fact they are beyond health insurance coverage in many provinces. In conclusion, the lack of diagnostic capacity, especially the ability to provide rapid diagnosis, is a major obstacle preventing proper, effective MDR-TB detection in China. In June 2019, the Chinese government announced Action Plan to Stop Tuberculosis (2019–2022). The plan aims to enhance laboratory capacity for MDR-TB diagnosis, requiring a DST coverage higher than 90% among bacteriologically confirmed cases by 2022. The strategy includes developing rapid molecular diagnostics for TB drug susceptibility and training procedures for associated laboratory staff, potentially allowing a significant acceleration in MDR-TB diagnosis.

In addition to case detection, treatment coverage is also essential to the effective control of MDR-TB. Multiple reasons lead to the difficulty of treatment enrollment, including economic hardship, loss to follow-up or death prior to treatment initiation, domestic migration caused by work and the misbelief that MDR-TB could be cured with DS-TB treatment. Whereas, economic hardship is the most common reason due to the high cost for medicines. While the expenses can be partially covered by health insurance, MDR-TB patients are still responsible for a considerable amount of treatment-related expenses that tend to be unaffordable for low-income patients. Findings from previous studies proved that 38% of MDR-TB patients experienced catastrophic healthcare expenses (more than 20% of annual household income) [33]. Consequently, it would be unrealistic to expect patients to bear the high-cost of MDR-TB care by themselves without risking catastrophic expenditures. Whereas, the decline in international donor funding now leaves it to the government to increase the amount of internal funding for the war against the MDR-TB epidemic.

The present study does show restrictions. For example, the general model was built on current estimates without taking new actions and interventions into consideration, such as policy changes, the introduction of new diagnostics, novel medicines, etc. In addition, some parameter values could not be identified accurately and thus, estimates based on several individual studies conducted in different years were used, which may affect the model’s accuracy. Therefore, alternative models projecting the MDR-TB epidemic should be considered and the corresponding result should be compared with the findings in the present study to gain a comprehensive understanding of the trends of TB/MDR-TB in China. Third, the constructed simple Markov model was unable to consider every detail of the transition process. In the present analysis, certain less influential parameters were simplified to perform the estimation, including the progression from DS-TB^+^ untreated to MDR-TB untreated and TB recurrence.

## Conclusions

The present study illustrated the challenges of MDR-TB confronted by China’s TB control program and provided unique insights into MDR-TB intervention. While the prevalence of DS-TB^+^ displayed a significant decrease, the MDR-TB epidemic would continue to grow rapidly. Improving MDR-TB detection and treatment coverage independently could reduce MDR-TB prevalence altogether, but was unlikely to halt the rise of MDR-TB. The results above indicated that enhanced interventions on promoting MDR-TB detection and treatment simultaneously would be imperative to stop the growing MDR-TB epidemic in China. Such enhanced interventions should involve increased emphasis on DST accessibility for all bacteriologically confirmed TB cases, rapid diagnosis, expanded health insurance coverage, and out-of-pocket payments exemption.

## Data Availability

All data generated or analyzed during the present study was included in this published article.

## Abbreviations

MDR-TB: Multidrug-resistant tuberculosis
DS-TB^+^: Bacteriologically confirmed drug-susceptible tuberculosis
TB: Tuberculosis
WHO: World Health Organization
RR-TB: Rifampicin-resistant tuberculosis
LTBI: Latent tuberculosis infection
DS-TB: Drug-susceptible tuberculosis
DS LTBI: Latent infection of drug-susceptible tuberculosis
MDR LTBI: Latent infection of multidrug-resistant tuberculosis
DST: Drug susceptibility test
MTB: *Mycobacterium tuberculosis*
